# Urinary incontinence developmental trajectories and risk predictors: a prospective study from pregnancy to 4 years after childbirth

**DOI:** 10.1186/s12889-025-24742-5

**Published:** 2025-10-09

**Authors:** Xiaojuan Wang, Minna Mao, Fang Xie, Rujia Zhao, Pingping Guo, Wei Zhang, Suwen Feng

**Affiliations:** 1 Women’s Hospital, Zhejiang University School of Medicine, No.1 Xue Shi Road, Hangzhou, Zhejiang Province 310006 China; 2 Zhejiang University School of Medicine, No.866 Yu Hang Tang Road, Hangzhou, Zhejiang Province 310058 China; 3 Hangzhou Wenhui School, No. 518 Jinqiao Road, Hangzhou, Zhejiang Province 310018 China

**Keywords:** Postpartum period, Pregnancy, Prospective studies, Risk predictor, Urinary incontinence trajectories

## Abstract

**Background:**

Urinary incontinence is a prevalent and bothersome health problem amongst women worldwide. Recognizing urinary incontinence trajectories and associated risk predictors are crucial for the prevention of urinary incontinence across the life course. Little was known about the developmental trajectories of urinary incontinence in adults. The study aims to identify the developmental trajectories of urinary incontinence from pregnancy to 4.5 years after childbirth and develop a dynamic nomogram based on the risk predictors of the incontinence trajectories.

**Methods:**

This was a long-term prospective study. A total of 1243 pregnant women were enrolled in late pregnancy and followed up at 6 to 8 weeks postpartum, one year and 4.5 years postpartum. Group-based trajectory modeling was applied to identify unrecognized trajectories of urinary incontinence from pregnancy to 4.5 years after childbirth. Logistic regression analysis with a backward stepwise process was conducted for risk predictor selection. Decision curve analysis was applied to assess the net benefit of the nomogram. Bootstrapping procedure with 1000 resamples was performed for internal validity.

**Results:**

A total of 1184 (95.3%) women who completed at least two follow-up assessments were included for trajectory analysis. Two distinct trajectories of urinary incontinence were identified with satisfactory model adequacy. Of the participants, 395 (33.4%) had persistently high risk of developing incontinence and 789 (66.6%) had low risk. Six risk predictors were associated with increased risk of developing high risk trajectory. Urinary incontinence before pregnancy was the strongest predictor (OR, 4.5; 95%CI, 3.0-6.5). A dynamic nomogram was developed by integrating the predictors, showing good predictive performance and clinical usability.

**Conclusions:**

One third of women were in persistently high risk group of urinary incontinence after childbirth. UI history before pregnancy, familial predisposition, vaginal birth, older age at first birth, greater pre-pregnancy BMI and living in rural areas were associated with increased risk of persistently high risk incontinence trajectory. Preventive efforts such as weight management before pregnancy and supervised pelvic floor muscle training could be made as early as possible.

**Supplementary Information:**

The online version contains supplementary material available at 10.1186/s12889-025-24742-5.

## Background

Urinary incontinence (UI) is a significant and distressing public health concern amongst women worldwide [1–3]. Large population-based studies indicated that 25–45% of adult women suffered from UI, and it was estimated that 13.6% of women would undergo UI surgery over their lifetime [4, 5]. The burden and health care demand will increase continuously over the next few decades as a result of population ageing [6]. UI can be prevented and there is consensus that screening individuals at high risk is the key for the prevention of future or persistent UI across the life course [7]. However, little progress has been made in the early prediction of UI.

The developmental process of UI across lifetime is dynamic and evolves over time, especially from pregnancy to the postpartum period [8, 9]. There was evidence that the prevalence of UI reached the first peak during late pregnancy and then decreased within one year postpartum [10]. Currently, studies regarding adult UI usually assumed that all participants followed a similar pattern over time, which oversimplified the complex developmental process of incontinence in clinical context [11]. Group-based trajectory modeling is developed to address this challenge, which can identify unrecognized distinct groups and provide the capability for identifying risk factors that predict these time-based progressions [12]. Jon et al. identified distinct trajectories of childhood bedwetting and daytime wetting using latent class analysis and found that different trajectories were associated with preschool sleep problems and adolescent bladder symptoms differentially [13, 14]. To date, the developmental trajectories of UI in adults remain unknown, let alone predictors of the trajectories.

Recognizing risk predictors of UI trajectories is crucial to screening women at high risk and supporting clinical decision making. Prediction model can not only identify predictors of UI trajectories but also give an estimated probability individually and accurately [15]. Currently, a few prospective risk prediction models of UI were developed with secondary data or with a very short-term follow-up, based on single observation of UI symptoms rather than dynamic trajectories over time [16–18]. As a result, no existing prediction tools for UI have been applied in clinical practice [7].There is an urgent need to develop a practical and parsimonious tool for predicting UI trajectories so that women at high risk could be prioritized for intervention.

The study aims to identify the developmental trajectories of UI from pregnancy to the postpartum period and develop a nomogram to recognize women at high risk of developing UI over time by integrating the risk predictors of the UI trajectories.

## Methods

### Participants

Pregnant women were consecutively enrolled in the obstetric wards of a tertiary maternity hospital in eastern China from January to June 2020 and followed up at 6 to 8 weeks postpartum, 1 year and 4.5 years postpartum. The inclusion criteria were: (1) aged 18 years or older; (2) term and singleton pregnancy; (3) willing to participate in the follow-up study. Participants were excluded from the study if they had the following conditions: (1) active urinary tract infection; (2) stillbirth; (3) fetus with congenital malformation; (4) severe comorbidity such as severe cardiopulmonary disease and kidney diseases.

### Measurements

Baseline data were collected with questionnaires when a pregnancy reached between 37 0/7 weeks and 41 6/7 weeks of gestation. Obstetrical data were obtained from medical records after childbirth. The place of residence (rural area or urban area) was reported by the participants and was verified through medical records. UI was assessed at baseline, 6 to 8 weeks postpartum, 1 year and 4.5 years postpartum using a standardized questionnaire. All data were collected by trained researchers. Baseline data and follow-up data were collected through a pencil and paper survey and an electronic questionnaire, respectively.

#### Outcome assessment

UI was measured with the International Consultation on Incontinence Modular Questionnaire-Urinary Incontinence Short Form, which was widely used to assess UI [19]. In this study, UI referred to the presence of any involuntary urinary leakage over the past four weeks based on the first question of the International Consultation on Incontinence Modular Questionnaire-Urinary Incontinence Short Form. Participants were considered to have UI if they ticked one with any complaint of urinary leakage. The Chinese version of the questionnaire was confirmed with good reliability and validity [20].

#### Candidate predictors

Fifteen candidate predictors were included for model development based on literature review and expert knowledge, consisting of age, education level, place of residence, job, menstrual status before pregnancy, pre-pregnancy BMI, family history of UI, medical history (childhood enuresis, gestational diabetes mellitus, history of urinary tract infection, UI before pregnancy) and obstetrical factors (age at first birth, birth mode, parity at baseline and baby birth weight). Amongst these predictors, age, pre-pregnancy BMI and age at first birth were continuous variables and the others were categorical variables.

### Statistical analysis

Sample size was calculated based on sample size calculation guidance for model development. Three parameters were needed including overall outcome proportion, the number of candidate predictors and C-statistic [21]. The overall outcome (high risk trajectory group) proportion was estimated to be 30%. The C-statistic was estimated to be 0.763 based on a previous study [16]. The number of candidate predictors was 15. Finally, a total of 686 participants were required to ensure statistical power.

Group-based trajectory modeling was applied to identify distinct trajectories of UI over time with the “traj” plugin in Stata 15.0. All available data were included for trajectory modeling under the assumption that data missing was at random. Bayesian information criterion (BIC) and model adequacy were used to select the best fitting model [12, 22]. Trajectory analysis involved a two-stage model selection process, which was a very practical approach [22]. First, we fitted the models from one to four group trajectories with follow-up time as a time scale, using the logistic model and a cubic trajectory function to determine the number of groups. Second, the focus was to determine the preferred order of the polynomial given the decision on number of groups. Possible orders of the polynomial of the two groups were fitted from zero order to cubic order and a separate model was ran for each potential combination of polynomial forms [22]. Finally, the optimal model was selected based on BIC value (the higher, the better) and model adequacy including the following criteria [12]: (1) the average of the posterior probabilities of group membership exceeded a minimum threshold of 0.7 for all groups; (2) a close correspondence between the probability of group membership and the group proportion; (3) the odds of correct classification was greater than 5 for all groups; (4) at least 30 individuals were included in each group. The characteristics between distinct trajectory groups were compared with an independent *t*-test and chi-square test.

Logistic regression analysis was conducted for predictor selection. The final model was determined by a backward stepwise process (*p* ≤ 0.1) with Akaike information criterion as the selection rule [23]. A dynamic nomogram was built based on the final model. The predictive performance of the nomogram was evaluated by discrimination and calibration, which were measured by the area under the receiver operating characteristic curve, Hosmer-Lemeshow test and calibration curve, respectively. Decision curve analysis was used to assess the net benefit of the prediction model [23]. Bootstrapping procedure with 1000 resamples was performed for internal validity [23]. Descriptive statistical analysis, logistic regression, the receiver operating characteristic curve, decision curve analysis and Hosmer-Lemeshow test were performed using Stata 15.0 software. Calibration curve, bootstrapping procedure and nomogram were done using the “rms” and “DynNom” packages in R software version 4.4.1. A *p*-value (two sided) less than 0.05 was considered statistically significant.

### Sensitivity analysis

The “traj” plugin in Stata provided full information maximum likelihood estimates by default to deal with data missing at random during the follow-up. We performed a sensitivity analysis to detect the impact of missing data during the follow-up on trajectory modeling. First, baseline characteristics between participants included in trajectory analysis and those excluded from trajectory analysis were compared. Second, we replicated trajectory analysis and logistic regression by including participants with complete follow-up data (*n* = 1038) to identify if UI trajectories as well as associated risk predictors were the same as those in the final model.

## Results

### Population characteristics

**Fig. 1:**
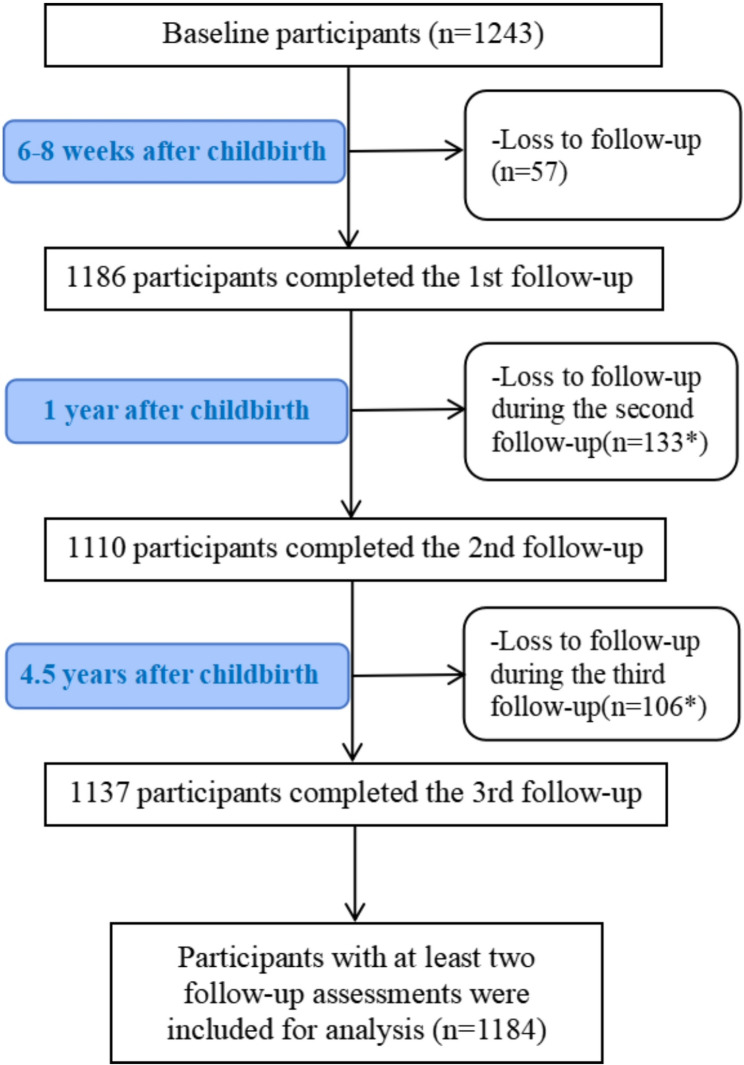
Flow chart of the study *All participants at baseline were contacted at each follow-up and some participants loss to follow-up provided data in later follow-ups

The flow chart of the study was shown in Fig. 1. A total of 1243 pregnant women were enrolled at baseline and 1184 (95.3%) women who completed at least two follow-up assessments were eligible for data analysis. The average age of the participants was (30.7 ± 4.0) years. Of the sample, more than 50% of the participants had bachelor or higher degree and 62.8% of the participants were primiparous.

### UI group trajectories

The optimal model with two distinct trajectory groups and cubic order was identified, which had higher BIC value (BIC=−2602.72) and satisfactory model adequacy. The average of the posterior probabilities of group membership were 0.92 and 0.80 for the low risk group and high risk group, respectively. There was a close correspondence between the probability of group membership and the group proportion. The odds of correct classification was 5.5 and 8.4 for the low risk group and high risk group, respectively. The fit statistics to determine the number of groups were shown in Table 1. Three best models (with the highest BIC values) of two and three UI trajectory groups were shown in Table 2. As indicated in Table 3, all parameter estimates of the optimal trajectory model were statistically significant.

**Table 1:** Fit statistics to determine the number of UI trajectory groups

Number of groups(order)	Fit statistics		
	BIC(*n* = 1184)	Probability of group membership	Group proportion
1(3)	−2748.59	100%	100%
2(33)	−2602.72	Group1 67.8% Group2 32.2%	Group1 66.6% Group2 33.4%
3(333)	−2614.31	Group1 31.9% Group2 54.0% Group3 14.1%	Group1 31.4% Group2 55.8% Group3 12.8%
4(3333)	−2630.94	Group1 23.6% Group2 20.1% Group3 42.2% Group4 14.1%	Group1 31.4% Group2 19.4% Group3 33.5% Group4 15.7%

*UI* urinary incontinence, *BIC* bayesian information criterion

**Table 2:** Three best models (with the highest BIC values) of two and three UI trajectory groups

Number of groups(order)	Fit statistics				
	BIC(*n* = 1184)	Probability of group membership	Group proportion	AvePP	OCC
2(33)	−2602.72	Group1 67.8% Group2 32.2%	Group1 66.6% Group2 33.4%	Group1 0.92 Group2 0.80	Group1 5.5 Group2 8.4
2(23)	−2608.79	Group1 54.9% Group2 45.1%	Group1 60.1% Group2 39.9%	Group1 0.85 Group2 0.90	Group1 4.6 Group2 11
2(32)	−2640.02	Group1 60.0% Group2 40.0%	Group1 59.9% Group2 40.1%	Group1 0.90 Group2 0.85	Group1 6.0 Group2 8.5
3(032)	−2601.36	Group1 16.6% Group2 65.8% Group3 17.6%	Group1 27.5% Group2 56.0% Group3 16.5%	Group1 0.54 Group2 0.89 Group3 0.80	Group1 0.2 Group2 4.2 Group3 18.2
3(312)	−2603.87	Group1 58.7% Group2 27.0% Group3 14.3%	Group1 56.1% Group2 31.4% Group3 12.5%	Group1 0.81 Group2 0.67 Group3 0.79	Group1 3.0 Group2 5.5 Group3 23.1
3(310)	−2604.51	Group1 54.2% Group2 42.1% Group3 3.7%	Group1 43.3% Group2 51.5% Group3 5.2%	Group1 0.94 Group2 0.78 Group3 0.65	Group1 13.3 Group2 4.9 Group3 44.6

*UI* urinary incontinence, *BIC* bayesian information criterion, *AvePP* the average of the posterior probabilities of group membership, *OCC* the odds of correct classification

**Table 3:** Parameter Estimation of the final trajectory model with two trajectories

Trajectory group	Parameter	Estimate	SE	Z value	*p* value
1 Low risk group	Intercept	−0.47266	0.10296	−4.591	< 0.0001
	Linear	−0.24999	0.02664	−9.384	< 0.0001
	Quadratic	0.00483	0.00061	7.872	< 0.0001
	Cubic	−0.00002	< 0.0001	−7.526	< 0.0001
2 Persistently high risk group	Intercept	1.47024	0.21895	6.715	< 0.0001
	Linear	−0.25148	0.03382	−7.435	< 0.0001
	Quadratic	0.00525	0.00074	7.142	< 0.0001
	Cubic	−0.00002	< 0.0001	−7.028	< 0.0001

**Fig. 2:**
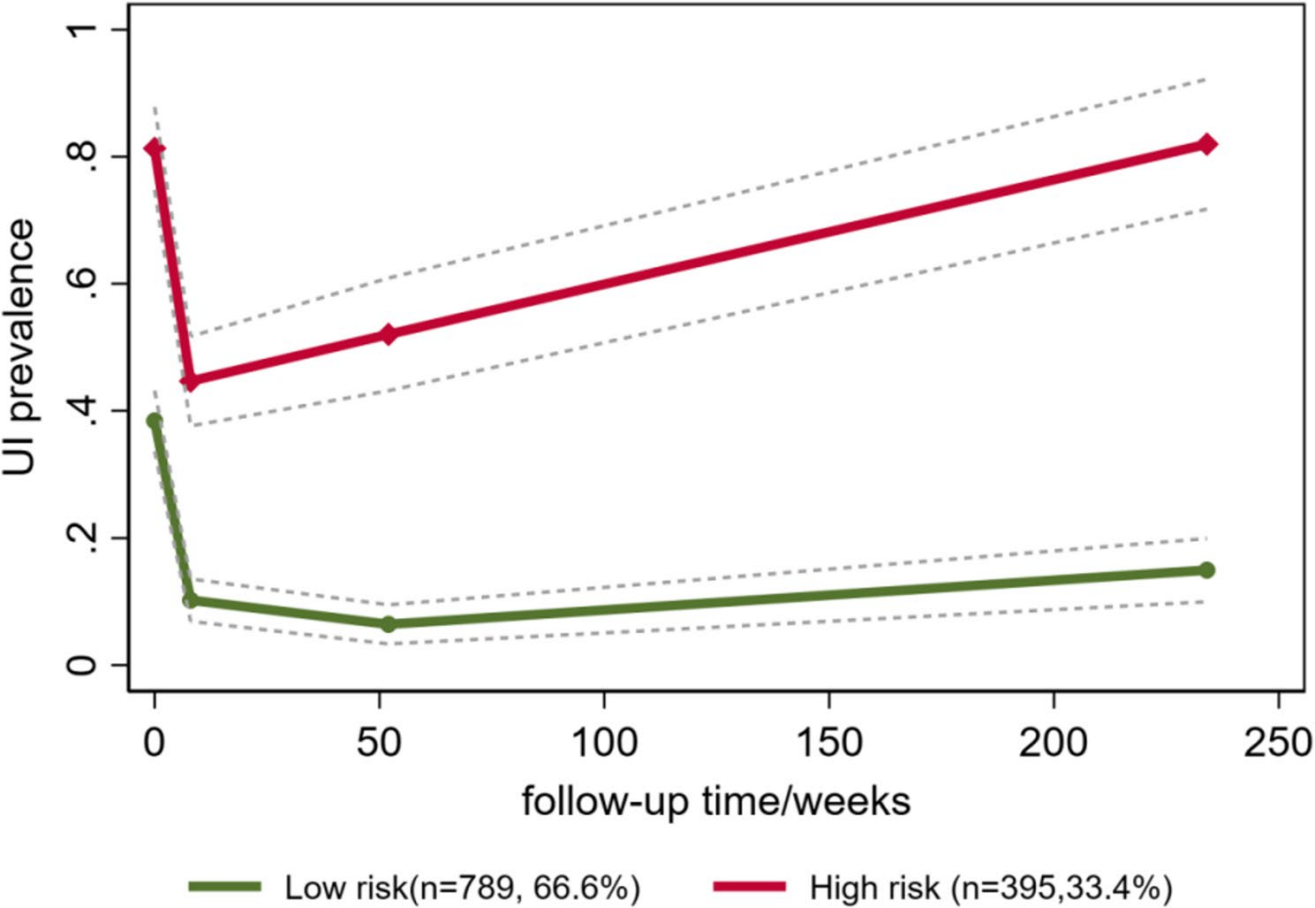
Trajectory groups of urinary incontinence over time

The optimal UI group trajectories were shown in Fig. 2. Participants in group 1 (low risk, *n* = 789, 66.6%) had moderate risk of developing UI in late pregnancy, with a rapid decline from late pregnancy to 6–8 weeks postpartum and remained relatively stable at low risk afterwards. Participants in group 2 (persistently high risk, *n* = 395, 33.4%) had high risk of developing UI in late pregnancy, followed by a steep decline to moderate risk until 6–8 weeks postpartum with a stable increase to high risk afterwards. At 6 to 8 weeks postpartum, the risk of developing UI in group 2 reached the lowest, which was still greater than the peak risk of group 1.

### Characteristics of the distinct trajectory groups

The characteristics of the participants by UI trajectory groups were shown in Table 4. Compared with participants in the low risk group, participants in the high risk group were older (28.7 years vs. 28.2 years, *p* = 0.038) when giving birth to the first baby, with greater pre-pregnancy BMI (21.4 kg/m^2^ vs. 21.0 kg/m^2^, *p* = 0.007). Participants in the high risk group were more likely to suffer from UI before pregnancy (23.0% vs. 7.1%, *p <* 0.001), report family history of UI (7.3% vs. 3.0%, *p* = 0.001) and history of urinary tract infection (18.7% vs. 13.8%, *p* = 0.027), and undergo vaginal birth (70.6% vs. 53.4%, *p <* 0.001). There was no statistically significant difference in gravidity and parity during the follow-up between the two groups.

**Table 4:** Characteristics of the participants by two trajectory groups

Variables	G1 Low risk (*n* = 789)	G2 Persistently high risk (*n* = 395)	*p* value
Age(years)	30.6 ± 4.0	31.0 ± 4.0	0.129
Education			
Junior college or below	278(35.3%)	147(37.2%)	0.568
Bachelor	405(51.3%)	190(48.1%)	
Master or above	106(13.4%)	58(14.7%)	
Place of residence			
Country	149(18.9%)	82(20.8%)	0.443
City	640(81.1%)	313(79.2%)	
Job			
Mental labor	694(88.0%)	344(87.1%)	0.667
Manual labor	95(12.0%)	51(12.9%)	
Menstrual status			
Regular	652(82.6%)	320(81.0%)	0.492
Irregular	137(17.4%)	75(19.0%)	
Pre-pregnancy BMI(kg/m^2^)	21.0 ± 2.7	21.4 ± 2.9	0.007
Family history of UI			
Yes	24(3.0%)	29(7.3%)	0.001
No	765(97.0%)	366(92.7%)	
Childhood enuresis			
Yes	65(8.2%)	42(10.6%)	0.175
No	724(91.8%)	353(89.4%)	
Gestational diabetes mellitus			
Yes	175(22.2%)	94(23.8%)	0.531
No	614(77.8%)	301(76.2%)	
History of urinary tract infection			
Yes	109(13.8%)	74(18.7%)	0.027
No	680(86.2%)	321(81.3%)	
UI before pregnancy			
Yes	56(7.1%)	91(23.0%)	< 0.001
No	733(92.9%)	304(77.0%)	
Age at first birth(years)	28.2 ± 3.6	28.7 ± 3.5	0.038
Birth mode ^a^			
Vaginal birth	420(53.4%)	279(70.6%)	< 0.001
Cesarean section	367(46.6%)	116(29.4%)	
Parity at baseline			
Primiparous	493(62.5%)	251(63.5%)	0.722
Multiparous	296(37.5%)	144(36.5%)	
Baby birth weight(g) ^a^			
<4000	745(94.7%)	366(92.7%)	0.171
≥ 4000	42(5.3%)	29(7.3%)	
Gravidity during follow-up ^b^			
0	575(78.2%)	309(81.7%)	0.170
≥ 1	160(21.8%)	69(18.3%)	
Parity during follow-up ^b^			
0	658(89.5%)	345(91.3%)	0.355
1	77(10.5%)	33(8.7%)	

*BMI* body mass index, *UI* urinary incontinence

^a^ Data of two participants were missing; Birth mode referred to the delivery mode of the first birth after enrollment

^b^ Data of 71 participants were missing due to dropout; Gravidity during follow-up referred to the number of subsequent pregnancies occurring after the index pregnancy; Parity during follow-up referred to the number of subsequent deliveries occurring between 6 weeks postpartum and 4.5 years postpartum

### Development of a nomogram for predicting UI trajectories

**Fig. 3:**
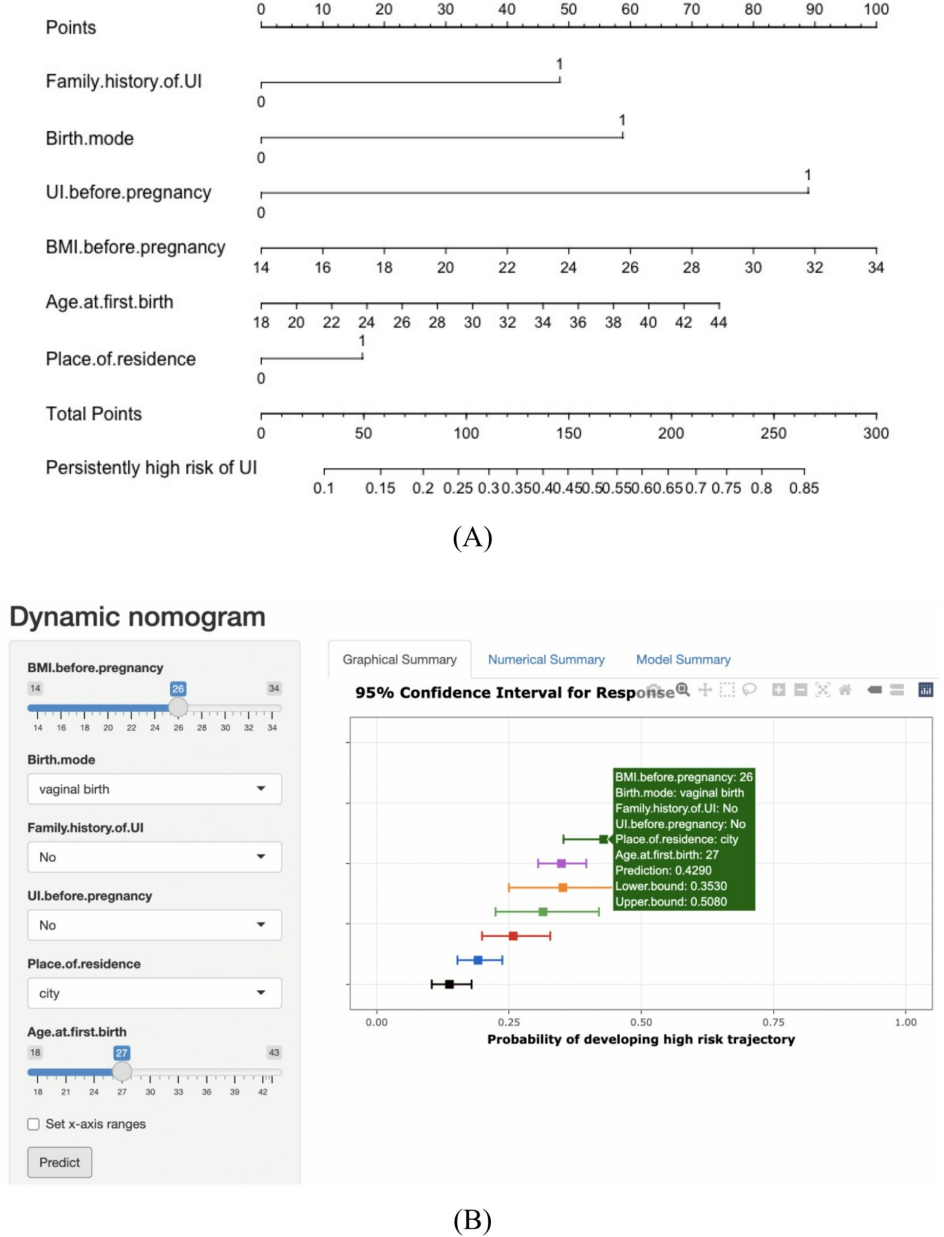
The nomogram (**A**) and dynamic nomogram (**B**) for predicting persistently high risk trajectory of urinary incontinence

As shown in Table 5, six risk predictors were left in the final model through logistic regression. Amongst these predictors, UI before pregnancy was the strongest predictor for developing persistently high risk trajectory of UI (OR, 4.5; 95%CI, 3.0-6.5), followed by birth mode and family history of UI. Women with greater pre-pregnancy BMI and older age at first birth were at greater risk of developinghigh risk trajectory. The prediction model was visualized by the nomogram in Fig. 3A. Besides, as shown in Fig. 3B, an online dynamic nomogram was developed to facilitate clinical usability (https://onlinenomo.shinyapps.io/DynNomo/).

**Table 5:** Logistic regression of the predictors for UI group trajectories

Intercept and variable	β	Odds Ratio(95%CI)	*p* value
Intercept	−4.753	--	< 0.001
Pre-pregnancy BMI (kg/m^2^)	0.084	1.1(1.0 to 1.1)	0.001
Family history of UI (yes vs. no)	0.815	2.3(1.3 to 4.0)	0.006
UI before pregnancy (yes vs. no)	1.493	4.5(3.0 to 6.5)	< 0.001
Birth mode (vaginal birth vs. cesarean section)	0.987	2.7(2.0 to 3.6)	< 0.001
Age at first birth (years)	0.048	1.0(1.0 to 1.1)	0.011
Place of residence (country vs. city)	0.276	1.3(1.0 to 1.8)	0.098

*UI* urinary incontinence, *BMI* body mass index

### Predictive performance and clinical use of the nomogram

The AUC was 0.682 in the training set and confirmed to be 0.671 via bootstrapping validation, demonstrating acceptable discrimination of the nomogram. No statistically significant difference was detected between observed and predicted probability of developing high risk trajectory in Hosmer-Lemeshow test and calibration curve (*p* = 0.576 and 0.987, respectively), showing satisfactory goodness of fit of the nomogram. The receiver operating characteristic curve and calibration curves of the nomogram were shown in Figure S1.

The decision curve analysis for the nomogram was presented in Figure S2, which showed that using this nomogram to predict the risk of developing high risk trajectory added more benefit than the treat-none or treat-all participants scheme when the threshold probability was between 15% and 80%.

### Sensitivity analysis

As shown in table S1, there was no statistically significant difference in baseline characteristics between participants included in trajectory analysis (*n* = 1184) and those who were excluded (*n* = 59). We replicated the trajectory analysis by including participants with complete data in three follow-up time points (*n* = 1038) with the same model selection criteria. As indicated in Figure S3, similar group trajectories were identified, including low risk group (*n* = 683, 65.8%) and persistently high risk group (*n* = 355, 34.2%). The predictors of UI trajectories analyzed with complete data were the same as the results in current analysis (Table S2).

## Discussion

This study identified distinct developmental trajectories of UI over time in adults, which provided novel and deep insights into the developmental pattern of UI from a brand new perspective with a long-term follow-up after childbirth. Unlike previous studies focusing on the average trend of UI, we applied group-based trajectory modeling to identify the unrecognized trajectories of UI over time in adults, which was crucial for identifying persistently high risk individuals. Further, an online tool was developed to screen women at persistently high risk with six predictors, which could facilitate the early intervention of high risk individuals.

As a data-driven approach, group-based trajectory modeling was particularly well suited to identify the underlying developmental heterogeneity in medicine and was applied in multiple mental and physical disorders, indicating remarkable heterogeneity in the developmental process of diseases over time [24–26]. Although there is a lack of trajectory modeling about UI in adults, two studies about childhood UI found that there were distinct trajectories for daytime wetting and bedwetting across childhood, amongst which persistent wetting trajectory group (bedwetting with daytime wetting until 9 years) indicated strongest relation to adolescent bladder symptoms [13, 14]. UI can be transient or persistent, which was obvious around childbirth [9]. In our study, the risk of developing UI in low risk group approached the lowest at 6 to 8 weeks postpartum and remained stable afterwards, which could be attributed to the recovery of incontinence when the pelvic floor organs returned to their original positions at 6 to 8 weeks postpartum [27]. For the high risk trajectory group, UI symptoms seemed unlikely to relieve over time and preventive efforts such as supervised pelvic floor muscle training should be made as early as possible.

Currently, clinical risk assessment of UI was crude, which was mainly based on clinical expertise and varied among clinicians. The nomogram provided a parsimonious and practical tool to screen women who were at high risk of developing UI and in need of intervention timely. In addition, informing women around childbirth about the probability of developing high risk trajectory could motivate them to comply with preventive strategies and improve their health outcomes [28, 29].

UI history played a significant role in the prediction of UI trajectory. We found that UI history before pregnancy was the strongest predictor of high risk trajectory group, which was consistent with the results in previous studies that UI before and during pregnancy were associated with UI afterwards [18, 30, 31]. Our finding supported that family history of UI contributed to the occurrence of incontinence in adults. There was evidence that familial predisposition of incontinence had far-reaching effect on bladder health, with greater risks among the relatives of women with severe incontinence symptoms [4, 18]. The mechanism underpinned the genetic effect might be attributed to bladder neck hypermobility, a heritable trait responsible for UI [4].

A wealth of studies detected a substantial difference in impact on short-term, long-term and persistent UI between vaginal birth and cesarean section [9, 32]. As one of the most important risk factors for UI, vaginal birth was also associated with an increased probability of high risk incontinence trajectory compared with cesarean section, further adding evidence to the causal role for vaginal birth. Pregnant women should be informed about the risks of different birth modes for bladder health when considering their birth modes [33]. Meanwhile, adverse outcomes for the mother and baby related to cesarean section such as abnormal placentation and increased risk of obesity in children should also be taken into account [34]. Our study suggested that older age at first birth was associated with high risk incontinence trajectory. A dose-response meta-analysis also concluded that older maternal age at first birth was associated with increased risk of UI postpartum [35].

Pre-pregnancy BMI was a significant predictor of high risk incontinence trajectory. Obesity was a well-accepted modifiable risk factor for UI. However, studies exploring the effect of pre-pregnancy BMI on UI postpartum were limited and the results were inconsistent [36, 37]. Lower rate of obesity before pregnancy and smaller sample size provided a plausible explanation for this discrepancy. Encouraging obese women with UI to lose weight in the general population was strongly recommended by guidelines [4, 38]. Our finding suggested that health care providers should pay attention to weight management before pregnancy, which could be beneficial for the prevention of persistently high risk UI trajectory after childbirth. We found that women living in rural areas were more susceptible to the high risk trajectory of UI, consistent with the finding in primiparous women [9]. The possible explanation was that women in rural areas were less accessible to maternal healthcare services compared with urban residents [39].

The study had limitations. First, there was loss to follow-up in this long-term study. Although the response rate was high, attrition bias was unavoidable. However, sensitivity analysis indicated that similar UI trajectories were identified using complete data and baseline characteristics between participants included in trajectory analysis and those who were excluded were comparable, denoting robustness of the results. Second, a few variables such as childhood enuresis were assessed with a self-reported questionnaire at baseline, which could introduce recall bias. Third, the participants were recruited from a tertiary maternal hospital. Validation of the nomogram in diverse settings should be conducted in the future. Fourth, there is still room for improvement in the predictive performance of the nomogram, additional predictors could be explored in further studies.

## Conclusion

In summary, there are two distinct UI trajectories among women after childbirth, which can be predicted through a dynamic nomogram. Pregnant women should be informed about their risks of developing high risk UI as early as possible so that preventive interventions such as weight management before pregnancy and supervised pelvic floor muscle training could be targeted accordingly.

## Supplementary Information

Supplementary Material 1.

## Data Availability

Reasonable requests could be submitted to the corresponding author via email (fengsw@zju.edu.cn).

## Abbreviations

UI: Urinary incontinence
ICIQ-UI SF: International Consultation on Incontinence Modular Questionnaire-Urinary Incontinence Short Form
BMI: Body mass index
