# Supplementary material for: Urinary incontinence developmental trajectories and risk predictors: a prospective study from pregnancy to 4 years after childbirth
Source: BMC Public Health. 2025 Oct 9;25:3433. doi: 10.1186/s12889-025-24742-5 (PMC12513054; doi:10.1186/s12889-025-24742-5)
Supplement: Supplementary file 1 — Supplementary Material 1. [file 12889_2025_24742_MOESM1_ESM.docx]

**Table S1** Baseline characteristics between participants included in trajectory analysis

and those who were excluded

| Variables | Participants included in analysis (n=1184) | Participants excluded from analysis (n=59) | *p* value |
| --- | --- | --- | --- |
| Age(years) | 30.7±4.0 | 30.1±4.1 | 0.240 |
| Education |  |  |  |
| Junior college or below | 425(35.9%) | 19(32.2%) | 0.739 |
| Bachelor | 595(50.2%) | 30(50.8%) |  |
| Master or above | 164(13.9%) | 10(16.9%) |  |
| Place of residence |  |  |  |
| Country | 231(19.5%) | 12(20.3%) | 0.875 |
| City | 953(80.5%) | 47(79.7%) |  |
| Job |  |  |  |
| Mental labor | 1038(87.7%) | 49(83.1%) | 0.296 |
| Manual labor | 146(12.3%) | 10(16.9%) |  |
| Menstrual status |  |  |  |
| Regular | 972(82.1%) | 52(88.1%) | 0.235 |
| Irregular | 212(17.9%) | 7(11.9%) |  |
| Pre-pregnancy BMI (kg/m^2^) | 21.0±2.7 | 21.0±3.0 | 0.688 |
| Family history of UI |  |  |  |
| Yes | 53(4.5%) | 3(5.1%) | 0.746 |
| No | 1131(95.5%) | 56(94.9%) |  |
| Childhood enuresis |  |  |  |
| Yes | 107(9.0%) | 6(10.2%) | 0.768 |
| No | 1077(91.0%) | 53(89.8%) |  |
| Gestational diabetes mellitus |  |  |  |
| Yes | 269(22.7%) | 15(25.4%) | 0.629 |
| No | 915(77.3%) | 44(74.6%) |  |
| History of urinary tract infection |  |  |  |
| Yes | 183(15.5%) | 8(13.6%) | 0.693 |
| No | 1001(84.5%) | 51(86.4%) |  |
| UI before pregnancy |  |  |  |
| Yes | 147(12.4%) | 8(13.6%) | 0.795 |
| No | 1037(87.6%) | 51(86.4%) |  |
| Age at first birth(years) | 28.4±3.6 | 28.6±3.6 | 0.704 |
| Birth mode ^a^ |  |  |  |
| Vaginal birth | 699(59.1%) | 38(65.5%) | 0.334 |
| Cesarean section | 483(40.9%) | 20(34.5%) |  |
| Parity at baseline |  |  |  |
| Primiparous | 744(62.8%) | 43(72.9%) | 0.118 |
| Multiparous | 440(37.2%) | 16(27.1%) |  |
| Baby birth weight(g) ^a^ |  |  |  |
| ＜4000 | 1111(94.0%) | 54(93.1%) | 0.775 |
| ≥4000 | 71(6.0%) | 4(6.9%) |  |

BMI, body mass index; UI, urinary incontinence.

^a^ Data of three participants were missing, birth mode referred to the delivery mode of the first birth after enrollment.

**Table S2** Logistic regression of the predictors for UI group trajectories (n=1038)

| Intercept and variable | β | Odds Ratio(95%CI) | *p* value |
| --- | --- | --- | --- |
| Intercept | -4.441 | -- | <0.001 |
| Pre-pregnancy BMI (kg/m^2^) | 0.072 | 1.1(1.0 to 1.1) | 0.006 |
| Family history of UI (yes vs no) | 0.802 | 2.2(1.2 to 4.2) | 0.012 |
| UI before pregnancy (yes vs no) | 1.548 | 4.7(3.1 to 7.2) | <0.001 |
| Birth mode (vaginal birth vs cesarean section) | 0.984 | 2.7(2.0 to 3.6) | <0.001 |
| Age at first birth (years) | 0.048 | 1.0(1.0 to 1.1) | 0.018 |
| Place of residence (country vs city) | 0.302 | 1.4(1.0 to 1.9) | 0.091 |

UI, urinary incontinence; BMI, body mass index.


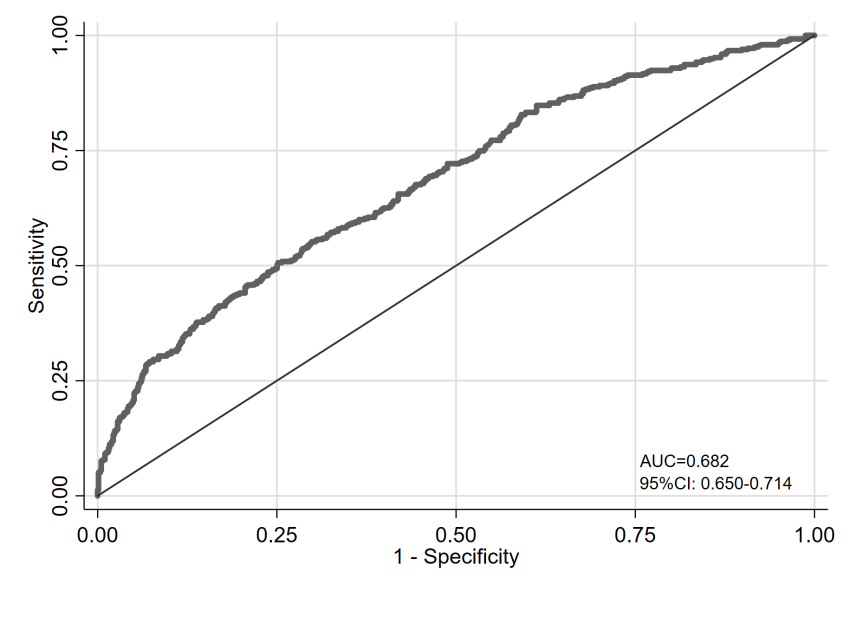


(A)


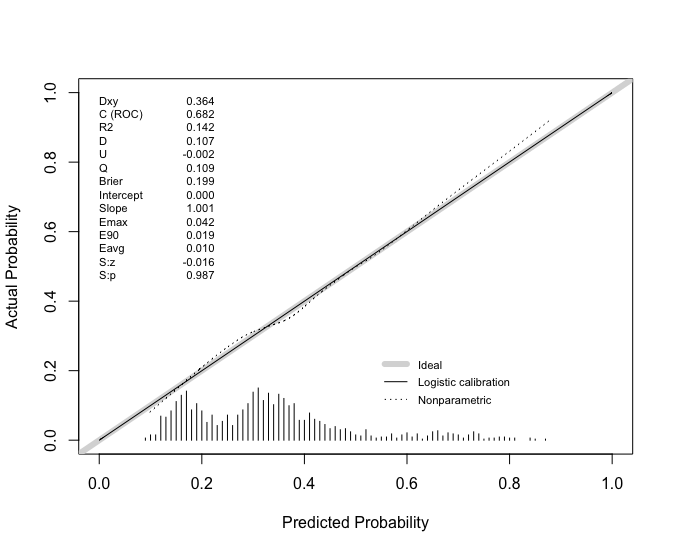


(B)


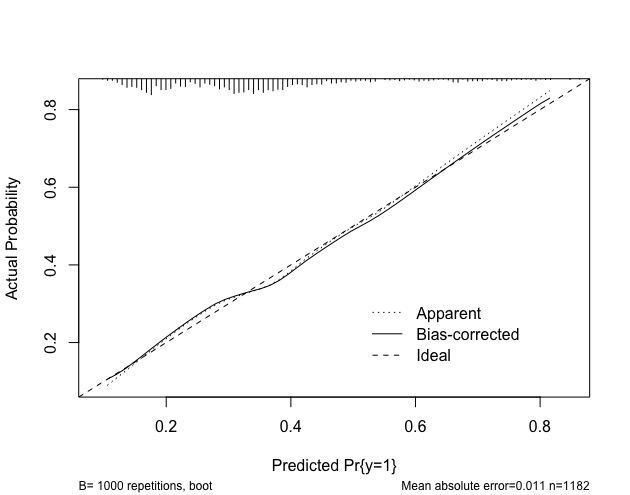


(C)

Fig. S1 The receiver operating characteristic curve of the nomogram in the training set (A). The calibration curve of the nomogram in the training (B) and internal validation sets (C).


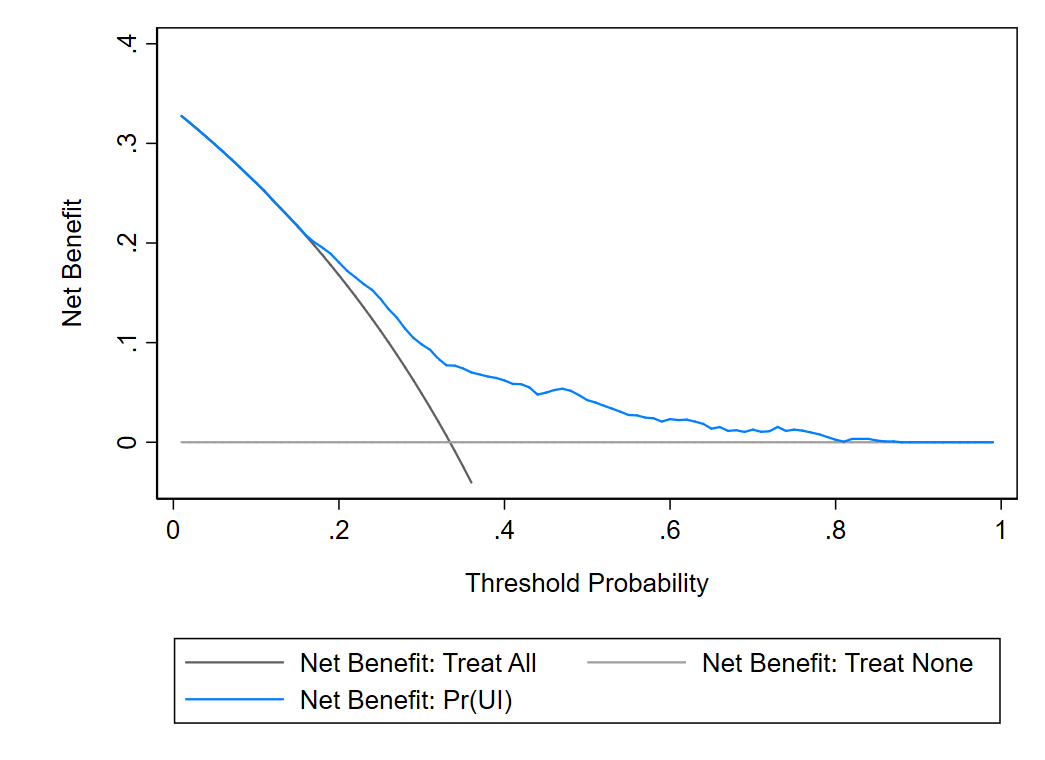


Fig. S2 The decision curve analysis for the nomogram


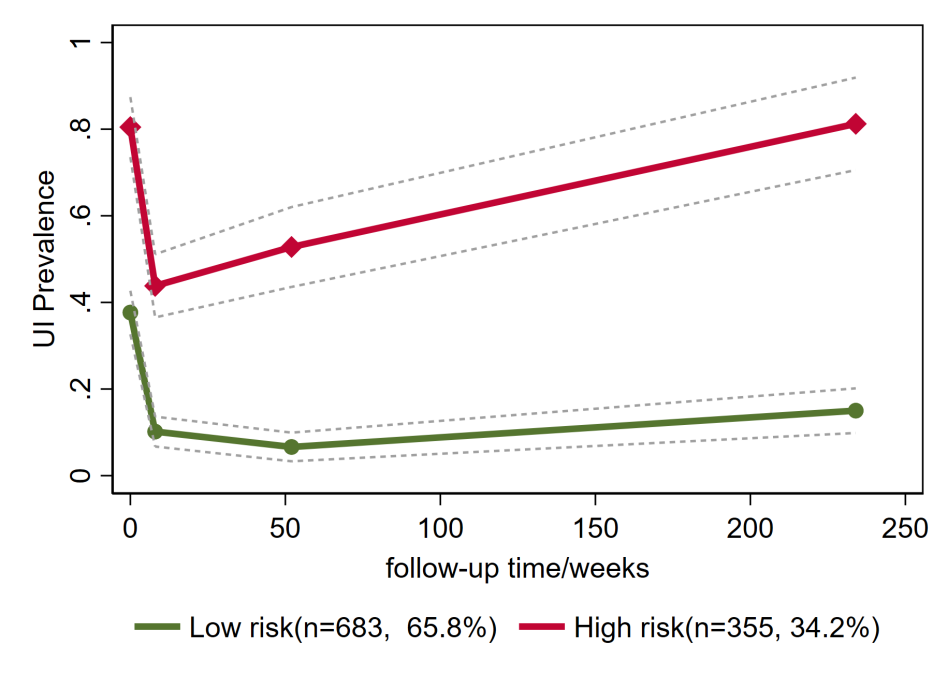


Fig. S3 Trajectory groups of urinary incontinence over time in participants with complete data in three follow-up time points (n=1038)
